# Diagnosis and treatment of right ventricular failure secondary to acutely increased right ventricular afterload (acute *cor pulmonale*): a clinical consensus statement of the Association for Acute CardioVascular Care of the European Society of Cardiology

**DOI:** 10.1093/ehjacc/zuad157

**Published:** 2023-12-22

**Authors:** Mattia Arrigo, Susanna Price, Veli-Pekka Harjola, Lars C Huber, Hannah A I Schaubroeck, Antoine Vieillard-Baron, Alexandre Mebazaa, Josep Masip

**Affiliations:** Department of Internal Medicine, Stadtspital Zurich, Birmensdorferstrasse 497, 8063 Zürich, Switzerland; Royal Brompton Hospital, National Heart & Lung Institute, Imperial College London, London, UK; Department of Emergency Medicine, Helsinki University Hospital, University of Helsinki, Helsinki, Finland; Department of Internal Medicine, Stadtspital Zurich, Birmensdorferstrasse 497, 8063 Zürich, Switzerland; Intensive Care Unit, Ghent University Hospital, Ghent, Belgium; Department of Intensive Care, Hôpital Ambroise-Paré AP-HP, Boulogne-Billancourt, France; Department of Anesthesia, Burn and Critical Care Medicine, AP-HP, Hôpitaux Universitaires Saint-Louis-Lariboisière, FHU PROMICE, INI-CRCT, and Université de Paris, MASCOT, Inserm, Paris, France; Research Direction, Consorci Sanitari Integral, University of Barcelona, Barcelona, Spain

**Keywords:** Right ventricular failure, Right heart failure, *Cor pulmonale*, Cardiogenic shock, Acute heart failure, Increased afterload, Pulmonary hypertension, Pulmonary embolism, ARDS

## Abstract

Acute right ventricular failure secondary to acutely increased right ventricular afterload (acute *cor pulmonale*) is a life-threatening condition that may arise in different clinical settings. Patients at risk of developing or with manifest acute *cor pulmonale* usually present with an acute pulmonary disease (e.g. pulmonary embolism, pneumonia, and acute respiratory distress syndrome) and are managed initially in emergency departments and later in intensive care units. According to the clinical setting, other specialties are involved (cardiology, pneumology, internal medicine). As such, coordinated delivery of care is particularly challenging but, as shown during the COVID-19 pandemic, has a major impact on prognosis. A common framework for the management of acute *cor pulmonale* with inclusion of the perspectives of all involved disciplines is urgently needed.

## Graphical Abstract

**Graphical Abstract:**
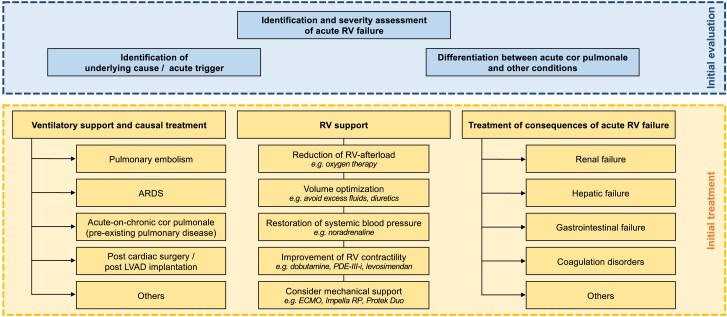
Management of acute *cor pulmonale*. ARDS, acute respiratory distress syndrome; LVAD, left ventricular assist device; PDE-III-I, phosphodiesterase-III inhibitors; RV, right ventricle; ECMO, extracorporeal membrane oxygenation.

## Purpose and aim

Acute right ventricular failure secondary to acutely increased right ventricular afterload (acute *cor pulmonale*) is a life-threatening condition that may arise in different clinical settings. Patients at risk of developing or with manifest acute *cor pulmonale* usually present with an acute pulmonary disease [e.g. pulmonary embolism, pneumonia, and acute respiratory distress syndrome (ARDS)] and are managed initially in emergency departments and later in intensive care units (ICUs). According to the clinical setting, other specialties are involved (cardiology, pneumology, internal medicine). As such, coordinated delivery of care is particularly challenging but, as shown during the COVID-19 pandemic, has a major impact on prognosis. A common framework for the management of acute *cor pulmonale* with inclusion of the perspectives of all involved disciplines is urgently needed.

The scope of this clinical consensus document is to provide an updated practical overview of the anatomical and physiological peculiarities of the right ventricle with particular focus on the pathophysiology of acute *cor pulmonale*, modern diagnostic approach to acute right ventricular failure including risk stratification, and contemporary management of acute *cor pulmonale* including specific treatment and mechanical circulatory and ventilatory support. The document includes the points of view of cardiologists, pulmonologists, emergency physicians, and intensivists involved in the care of these patients and focuses on the acute management occurring in emergency departments and intensive cardiovascular care units.

## Methods

The topic of this clinical consensus document was defined during a meeting of the Acute Heart Failure study group of the Acute CardioVascular Care Association of the European Society of Cardiology. Invited authors were assigned to one or more topics before, according to their field of expertise. Authors provided a written paragraph and the relevant literature related to their topic. The consolidated document was then discussed among all authors, and consensus was achieved on the relevant messages to be included in the manuscript. If there were any areas of uncertainty or controversy, the topic was discussed until a consensus was reached. The literature searches were updated, and the agreed views were finalized as the clinical consensus document was written and revised.

## Clinical vignette

A 50-year-old woman presented to the emergency department due to worsening dyspnoea and diaphoresis. Initial assessment revealed haemodynamic instability: blood pressure (BP) 75/40 mmHg, heart rate (HR) 125 b.p.m., and pulse oximetry oxygen saturation (SpO_2_) 80%, despite receiving oxygen therapy with a flow of 15 L/min. The patient was tachypnoeic, with respiratory rate (RR) 40–50/min, febrile (39.2°C), with extensive pulmonary wheezing, elevated jugular venous pressure, warm extremities, and no peripheral oedema. Blood lactate was 4.5 mmol/L, defining a Society for Cardiovascular Angiography and Interventions (SCAI) SHOCK stage C. Despite haemodynamic support with norepinephrine, the patient deteriorated, requiring endotracheal intubation and transfer to the ICU. Urgent echocardiography demonstrated a hyperdynamic left ventricle (LV); dilated, hypertrophic right ventricle (RV) with reduced systolic function; and moderate tricuspid regurgitation (TR), with a peak gradient of 90 mmHg. Despite haemodynamic support with norepinephrine (1 mcg/kg/min) and milrinone (0.15 mcg/kg/min), progressive instability and hypoxaemia developed (SCAI SHOCK stage D). Veno-arterial extracorporeal membrane oxygenation (VA-ECMO) was instituted as a bridge to recovery. Further investigations revealed an influenza A infection triggering a status asthmaticus and acute RV failure in a patient with pre-existing HIV-associated pulmonary arterial hypertension (PAH). Over the course of several days, respiratory and haemodynamic improvement was observed, VA-ECMO weaning was achieved, and the patient was discharged from the ICU.

## Anatomical and physiological particularities of the right ventricle

The anatomy of the RV is unique and adapted to fulfil its physiological functions, i.e. moving systemic blood through the lungs whilst maintaining an adequate LV preload.^1^ The RV is more compliant and under normal circumstances slightly larger (∼10–15%) than the LV, which accommodates large variations in venous return without altering end-diastolic pressure. The RV anatomy comprises the inlet with the tricuspid valve, chordae tendineae, at least three papillary muscles, the trabeculated apex, and the infundibulum (a muscular structure supporting the pulmonary valve). As pulmonary pressures are much lower than systemic, the RV needs fewer muscle fibres to achieve the same stroke volume as the LV and is therefore significantly thinner-walled. This anatomical peculiarity is responsible for the high sensitivity of the RV to changes in afterload. Rapid rises are poorly tolerated, leading to ventricular dilatation, leftward shift of the interventricular septum, and marked reduction in stroke volume, resulting in LV compression and insufficient filling, systemic hypotension, and shock.^2^ In most individuals, the right coronary artery perfuses the RV free wall and posterior part of the interventricular septum, whilst the left anterior descending artery perfuses the apex and the anterior part of the septum. Due to low intracardiac systolic pressures, RV myocardial perfusion occurs during both systole and diastole. Because of its thinner wall and higher dependence on coronary perfusion pressure, RV perfusion is more vulnerable to increases in wall tension (increased intramural pressure) and systemic hypotension.^3^ Therefore, in case of rapid increases in pulmonary pressures, RV overload occurs (e.g. acute *cor pulmonale*), and the combination of elevated RV wall tension and insufficient LV preload with systemic hypotension may precipitate RV myocardial ischaemia and dysfunction.

Despite differences in systolic contraction patterns (LV more circumferential, RV more longitudinal), both ventricles share septal function, with up to 40% of RV systolic function dependent on septal contraction.^4^ The interventricular septum also affects ventricular interdependence. Here, excessive volume and/or pressure loading dilates the RV leading to leftward shift of the septum and resulting in LV compression and reduction in stroke volume, because both ventricles ‘compete’ for space within the pericardium.^1^

## Pathophysiology of acute right ventricular failure

As no universally agreed definition of RV failure exists, different incidences of RV failure are reported according to the diagnostic tools used. Briefly, RV failure has been defined as acute RV dilation; the presence of impaired echocardiographic parameters of RV systolic function, i.e. tricuspid annular plane systolic excursion (TAPSE); and/or when RV dilation leads to significant systemic congestion with organ failure.^5^ This last definition would suggest the need for measuring central venous pressure (CVP) and organ function in combination with echocardiography. These different diagnostic approaches potentially lead to heterogeneous patient populations in clinical studies.^6,7^

An isolated increase in RV afterload may result from proximal obstruction of the pulmonary circulation, as in pulmonary embolism, or by a more distal obstruction at the level of the pulmonary capillaries, as observed in ARDS. In the latter situation, ventilator settings and blood gases contribute to RV failure besides the pulmonary pathology.^8^ In case of chronically increased RV afterload (e.g. chronic pulmonary hypertension), RV adaptation may become unbalanced by an acute intercurrent event such as infection, arrhythmia, or embolism (acute-on-chronic *cor pulmonale* pattern), as in the Clinical vignette (see above).

Three other main mechanisms of RV failure can be observed in critically ill patients.^9^ The first includes reduction in systolic function related to myocardial ischaemia from acute coronary syndrome (ACS), accounting for 5% of patients admitted for cardiogenic shock (CS), in turn representing 14–16% of ICU admissions.^6,10^ The second includes myocardial injury due to sepsis; septic cardiomyopathy affecting both ventricles is a combination of systolic dysfunction due to cytokines release and increased RV afterload due to mechanical ventilation; according to the definition used, it was reported between 23% and 48% of septic patients.^11–13^ The third includes RV volume as observed in TR and/or pulmonary regurgitation. Finally, RV failure may occur after cardiac surgery or in the context of LV assist device implantation.^14,15^

## Diagnosis of acute *cor pulmonale*

### Clinical diagnosis

Initial evaluation is based on clinical history and physical examination. The history of the patient’s present illness should raise suspicion of acute RV failure, considering several possible aetiologies [ARDS, (invasive or non-invasive) mechanical ventilation, post-operatively after cardiac surgery, LVAD implantation, or acute pulmonary embolism]. Differential diagnoses such as pericardial tamponade, acute right ventricular myocardial infarction, and pneumothorax may have similar clinical presentation and should be considered. In acute *cor pulmonale*, jugular vein distension is common and reflects RV dysfunction severity rather than volume status. Overt congestion with ascites and peripheral oedema is uncommon in the setting of *de novo* acutely increased RV afterload.^16^ Specific aetiologies of *cor pulmonale* can present with more disease-specific symptoms suggesting the diagnosis, e.g. in acute pulmonary embolism chest pain, dyspnoea, and/or syncope, might be present. An electrocardiogram (ECG) is of added value and may show changes due to RV dilation, with high specificity although sensitivity is low (*Table 1*).^17^ Patients with acute RV failure may develop CS (as in the Clinical vignette, see above), which is characterized by signs and symptoms of hypoperfusion and increased serum lactate levels (*Table 1*).^23,24^ Like CS due to acute LV failure, RV shock can be classified using the SCAI SHOCK stages, which may guide escalation and de-escalation of therapies.^25^

**Table 1:** Diagnosis and severity parameters of acute *cor pulmonale*

	Clinical parameters	Biochemical parameters	Echocardiographic parameters
RV stress	ECG signs of acute RV failure P pulmonale (P in II >2.5 mm) Sinus tachycardia or atrial fibrillation/flutter Right bundle branch block Right axis deviation S1q3T3 pattern	↑ High-sensitive troponin I or T	Systole–diastole septal shift: LV D-shaping and paradoxical septal wall motion RV/LV end-diastolic diameter ratio > 0.6 = moderate; ratio > 1.0 = severe RV dilation RV basal diameter > 42 mm^18^ McConnell’s sign (preserved apical RV contraction, mid free wall RV segment akinesia) Peak systolic velocity of TR jet > 2.8–3.5 m/s Pulmonary acceleration time (PAT) < 100 ms 60/60 sign (peak systolic TR gradient <60 mmHg; PAT < 60 ms) in case of acute pulmonary hypertension^16^ RA dilation, interatrial septum bulging to left atrium
RV dysfunction	Jugular venous distension	↑ NT-proBNP/BNP	TAPSE < 17 mm, systolic S′ velocity of tricuspid annulus < 9.5 cm/s by TDI, RV fractional area change < 35% Global longitudinal RV strain (−20%)^19^ Dilated inferior vena cava (>21 mm), respiratory variation <50%
Cardiogenic shock	Cold or mottled extremities Confusion/altered mental state Pulsus paradoxus in spontaneously breathing patients Low pulse pressure Tachycardia (>100 b.p.m.) Oliguria (<0.5 mL/kg/h) +/− Hypotension	↑ Lactate Metabolic acidosis Acute liver injury (transaminases, bilirubin, prolonged prothrombin time) Acute kidney injury (↑ serum creatinine, urea) SvO_2_↓/pCO_2_-gap > 6 mmHg	VTI_LVOT_ ≤ 13 cm^20^ Cardiac index ≤ 2.2 L/min/m²^21^ MV inflow variation ≥ 25%
Disease-specific: PE	PESI score^22^ Signs of deep venous thrombosis	↑ D-dimers	Thrombus in RA or proximal pulmonary arteries (requires TEE)

RV, right ventricle; PE, pulmonary embolism; b.p.m., beats per minute; ECG, electrocardiogram; PESI, pulmonary embolism severity index; VTI, velocity time integral; LVOT, left ventricular outflow tract; LV, left ventricle; TR, tricuspid regurgitation; TAPSE, tricuspid annular planar systolic excursion; TDI, tissue Doppler integral; RA, right atrium.

### Biochemistry/biomarkers

An arterial blood gas including blood lactate is mandatory in the initial management of the patient to determine shock states and their severity. Kidney and liver function should also be assessed for impairment. Venous congestion is the main trigger for reduced organ function, sometimes accompanied by inadequate cardiac output.^26,27^

Natriuretic peptides (BNP or NT-proBNP) are elevated in case of acute RV failure. Elevated high-sensitive troponin can reveal myocardial injury due to increased RV afterload. Both are associated with worse prognosis.^22,28^ When pulmonary embolism (PE) is suspected, D-dimers < 500 µg/L exclude this diagnosis with a sensitivity ≥ 95% in combination with low or intermediate pre-test probability, but false-positive results are frequent in critically ill patients. Alternatively, an age-specific cut-off can be used (age × 10 mg/L, for patients aged > 50 years).^22^

### Imaging

**Figure 1:**
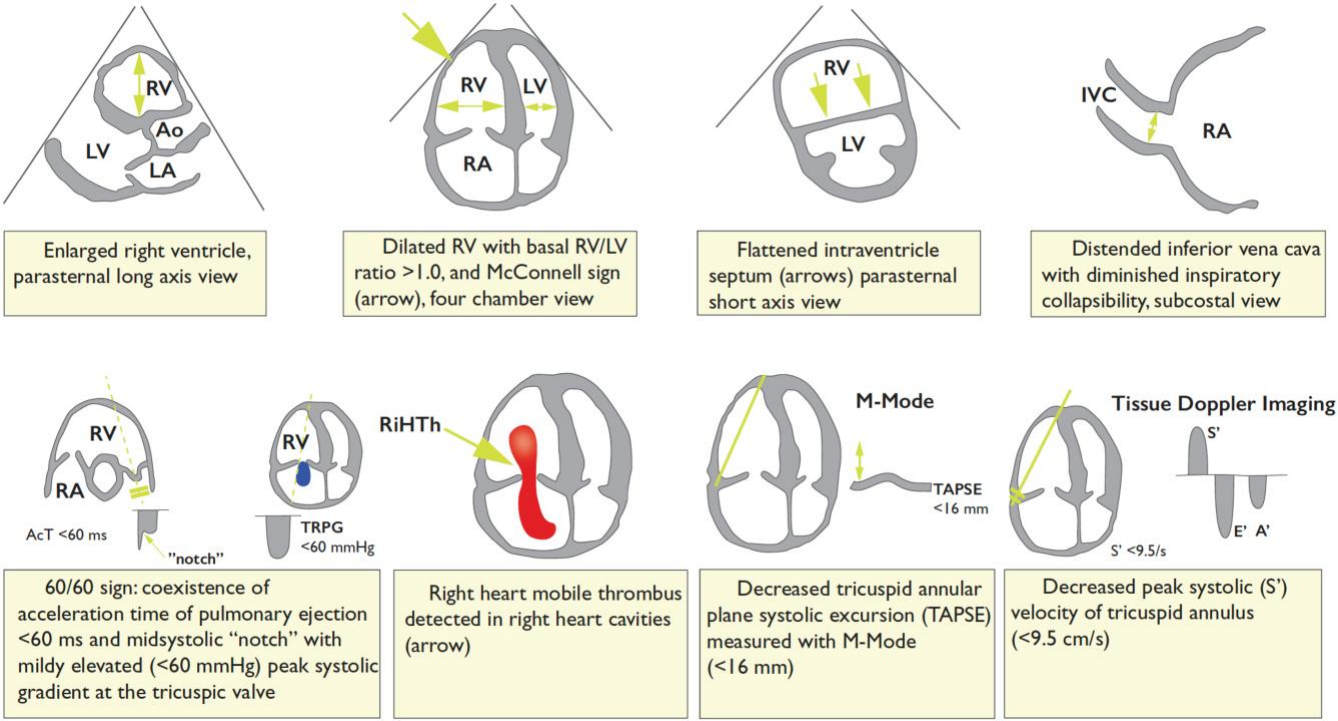
Echocardiographic assessment of the right ventricle. A′, peak late diastolic (during atrial contraction) velocity of tricuspid annulus by tissue Doppler imaging; AcT, right ventricular outflow Doppler acceleration time; Ao, aorta; E′, peak early diastolic velocity of tricuspid annulus by tissue Doppler imaging; IVC, inferior vena cava; LA, left atrium; LV, left ventricle; RA, right atrium; RiHTh, right heart thrombus (or thrombi); RV, right ventricle/ventricular; S′, peak systolic velocity of tricuspid annulus by tissue Doppler imaging; TAPSE, tricuspid annular plane systolic excursion; TRPG, tricuspid valve peak systolic gradient. Reproduced with permission from Konstantinides *et al.*^22^

Comprehensive transthoracic echocardiography (TTE) is the primary imaging technique to assess RV anatomy and function in the acute setting.^17^ Increased RV afterload will lead to RV dilation and systole–diastole septal shift causing a D-shaped LV.^16^ The RV function can be assessed by TAPSE, by tissue Doppler imaging (TDI) measuring systolic S′ velocity of tricuspid annulus and fractional area change of the RV (*Figure 1*).^16^ Global RV longitudinal strain using speckle tracking with a value of −20% is highly predictive of RV dysfunction (*Table 1*).^19^ An elevated gradient between RV and RA or a short pulmonary acceleration time (<100 ms) reflects high pulmonary artery (PA) pressures.^16^ The inferior vena cava is dilated and shows decreased respiratory variation (<50%). None of the echo parameters is specific to any cause, except in case a mobile thrombus is visualized.

Chest X-ray should be performed to unravel underlying pulmonary disease contributing to RV dysfunction.^18^ Computed tomography pulmonary angiography (CTPA), a CT scan of the chest with IV contrast, helps to detect a pulmonary cause of the increased RV afterload and assesses RV diameters in comparison with the LV. Cardiac MRI (CMR) is the gold standard to assess detailed RV function and anatomy but is seldom used in the acute phase.^17,18^ Computed tomography coronary angiography (CTCA) may be useful and could serve as an alternative for CMR to evaluate RV function by using a 3D volume segmentation tool.^29^

## Haemodynamic monitoring in acute *cor pulmonale*

Invasive haemodynamic monitoring provides additional parameters to assist the diagnosis of acute *cor pulmonale* and is essential for timely recognition of rapid changes in the haemodynamic status and for assessing the response to treatment.^30^ In patients with severe acute *cor pulmonale* with shock, arterial and central venous lines are standard of care.^31^ Central venous oxygen saturation as well as Pv-aCO_2_-gap could be derived and provide information about the perfusion status. In more complex cases, haemodynamic assessment with PA catheter provides a more exhaustive haemodynamic profile which assists the understanding of the aetiological mechanisms (including a post-capillary, left-sided component of RV afterload) and could help to tailor pharmacological and fluid management.^32^ Valuable parameters indicating relevant RV failure include elevated CVP > 15 mmHg, discordant right-to left filling pressures [right atrial pressure to pulmonary capillary wedge pressure ratio (RAP/PCWP) >0.8], low PA pulsatility index (PAPi) ≤ 1.85, and low RV stroke work index < 0.25–0.30 mmHg * L/m^2^^30^

In case of severe TR, PA measurements should be interpreted cautiously. Due to ventricular interdependence, tools that use pulse pressure variation and contour wave analysis are unreliable to assess volume status. Serial echocardiography determining right- and left-sided filling pressures and cardiac output is a good alternative to monitor patients with acute RV failure.^31^

## Oxygen and ventilatory support in acute *cor pulmonale*

Many of the clinical scenarios leading to acute *cor pulmonale* present with significant hypoxaemia, and therefore, oxygen and often ventilatory support is frequently needed. However, positive pressure ventilation can elevate pulmonary vascular resistance (PVR) and intrathoracic pressures, reducing venous return and consequently RV preload.^33^ The addition of positive end expiratory pressure (PEEP) extends these effects throughout the respiratory cycle, further reducing venous return and RV preload.^33, 34^ Hypoxaemia, hypercarbia, and acidaemia independently produce pulmonary vasoconstriction, and when combined, they have a synergistic effect.^33^ In chronic lung diseases, persistent hypoxaemia may cause vascular remodelling, growing the muscular layer, and narrowing the small pulmonary arteries. Both vasoconstriction and remodelling increase PVR and RV afterload.

**Figure 2:**
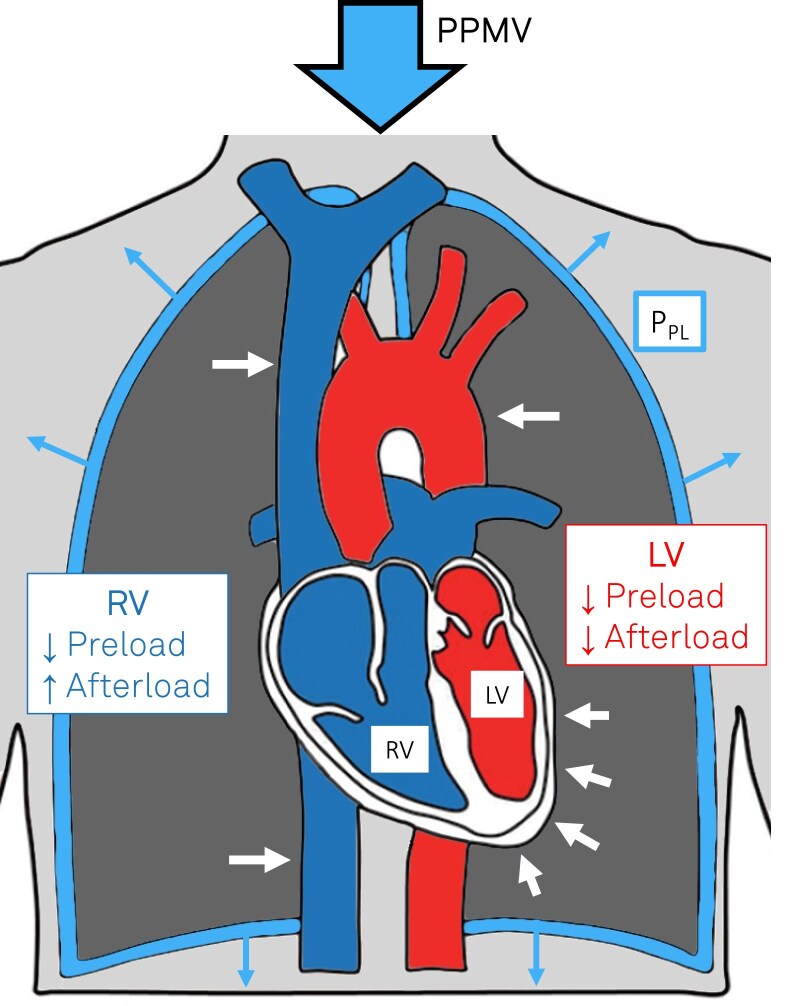
Effects of mechanical ventilation on cardiac function. The RV preload is inversely related to intrathoracic pressure. During positive pressure mechanical ventilation (PPMV), pleural pressure (*P*_PL_) rises, and RV preload falls and produces a detectable reduction in cardiac output. Positive pressure mechanical ventilation produces also an increase of RV afterload. In the other hand, PPMV reduces the preload and afterload of the left ventricle. Blue arrows, outward chest wall expansion by PPMV; white arrows, effect of positive PPL on intrathoracic vasculature and left ventricle. Modified from Cortes-Puentes *et al.*^35^

Effects of mechanical ventilation on cardiac function are depicted in *Figure 2*.

### Ventilation in specific scenarios

#### Acute respiratory distress syndrome

Around 20–30% of patients with moderate to severe ARDS may develop acute *cor pulmonale*.^8^ The increase in PVR is mainly due to the combination of persistent hypoxic/hypercapnic pulmonary vasoconstriction, lung inflammation affecting microcirculation and microthrombi formation, and ventilator-induced lung injury (VILI) (extremes of lung volume and imbalance between overdistension and recruitment). Prevention/mitigation manoeuvres include protective ventilation with low tidal volumes (6 mL/kg), optimized PEEP (initially <10 cmH_2_O appears reasonable even in the absence of scientific evidence), and control of the plateau pressure < 27 cmH_2_O or driving pressure < 17 cmH_2_O, avoiding significant hypercarbia (PaCO_2_ < 60 mmHg) and prompting to early prone position, if PaO_2_/FiO_2_ < 150mmHg.^36^ Prone position is one of the best manoeuvres to unload RV in ARDS.^37^ In selected patients, prone positioning extends the recruited and aerated areas of the lung, resulting in a more homogeneous ventilation, reducing V/Q mismatch thereby improving oxygenation and hypercapnia, and reducing pulmonary vascular tone, driving pressures, and VILI. All of these actions lead to a decrease in the RV afterload, with the potential to improve RV function. Although physicians may have concerns regarding proning unstable patients, when the instability is primarily secondary to RV dysfunction, haemodynamic parameters and circulatory failure may improve.^38^ In addition, it must be considered that prone positioning has demonstrated to reduce mortality in severe ARDS.^39^

#### High-risk pulmonary embolism

The treatment of hypoxaemia in acute RV failure due to acutely increased RV afterload should be considered in a stepwise manner, avoiding positive pressure ventilation where possible. Mechanical ventilation has shown to be associated with a three-fold higher risk of mortality in patients with high-risk PE.^40^ Therefore, oxygen therapy via high-flow nasal cannula should be initially used in hypoxic patients refractory to conventional oxygen therapy. For non-responders, cautious low-level of non-invasive ventilation would be the first option to minimize the impact on the RV. Intubation should be avoided, when possible, because many sedative agents may exacerbate haemodynamic instability. Even when mechanical ventilation is started, the correction of hypoxaemia in many cases would not be possible without simultaneous pulmonary reperfusion.^22^

#### Acute-on-chronic *cor pulmonale*

These patients need similar approach, avoiding or minimizing the impact of positive pressure on a failing RV with chronic PH. Some authors have proposed to perform awake intubation after nebulization of local anaesthesia, supported with inotropic–vasoactive drugs and inhaled vasodilators during the procedure.^41^ Due to the difficulties in the management of these patients, it is advisable to transfer them to specialized centres.

## Medical treatment of acute *cor pulmonale*

Effective treatment of RV failure requires a rapid restoration of adequate arterial blood pressure. To achieve this goal, volume optimization and frequently treatment with vasopressors and/or inotropes are needed.

### Volume optimization

Some patients with RV failure may be fluid responders, but in the majority of patients, fluid administration may be harmful and should be considered with the utmost caution. Volume loading has the potential to overdistend the right heart and subsequently increase wall tension, worsen ventricular interdependence, impair LV filling, aggravate TR, and ultimately reduce systemic cardiac output. Cautious volume loading guided by echocardiography, central venous or PA pressure monitoring, and/or cardiac output measurements may be appropriate if arterial hypotension is present without signs of elevated filling pressures. In contrast, diuretics may be indicated in patients with RV failure who present with signs of venous congestion.^16^

### Vasopressor and inotrope treatment

In patients with acute RV failure and haemodynamic instability or shock, vasopressors and/or inotropes are indicated to restore organ perfusion.^6^ Vasopressors are first indicated in case of hypotension^16^; they increase peripheral resistance and correct organ perfusion (including cardiac) by improving arterial blood pressure. Noradrenaline can improve systemic haemodynamics and coronary perfusion with minimal effect on PVR.^42^ Data for vasopressin are lacking in acute RV failure.^43^

In case of inadequate cardiac output, dobutamine, phosphodiesterase-III (PDE-III) inhibitors, and levosimendan may improve RV contractility and restore cardiac output.^44^ Dobutamine has a dose-dependent positive inotropic effect mediated through β-receptors on cardiomyocytes. Adrenergic stimulation increases the production of intracellular cyclic adenosine monophosphate (AMP) which leads to increased intracellular calcium concentration. Phosphodiesterase-III inhibitors exert their positive inotropic effect, decreasing the intracellular breakdown of cyclic AMP. In case of RV dysfunction, the use of inotropes should ideally be confirmed by measures of inadequate cardiac output despite restoration of blood pressure. Because of potential harmful effects, inotrope use should be limited to low cardiac output and be stopped as early as possible. No data support differences among inotropes in RV dysfunction.

## Specific treatments for the underlying cause of acutely increased right ventricular afterload

In the previously healthy individual, the most common cause of acute RV failure is high-risk pulmonary embolism.^17^ In addition to anticoagulation and supplementary oxygen, more aggressive management is reserved for patients presenting with shock, i.e. with haemodynamic compromise and cardiopulmonary instability.^45^ Here, systemic fibrinolysis offers an effective treatment option using recombinant tissue plasminogen activator (rtPA, alteplase, Actilyse®) in a dose of 100 mg over 2 h, of which 0.6 mg/kg are given as loading dose over 15 min. In patients with a body weight < 65 kg, the maximum dose should not exceed 1.5 mg/kg. There is no need to withhold unfractionated heparin during the administration of rtPA. Procedural success in terms of outcome improvement is potentially limited by complications, including bleeding and (in the event of cardiac arrest/profound hypotension) hypoxic brain injury. It remains matter of debate whether empirical thrombolysis should be already applied in patients with out-of-hospital cardiac arrest when pulmonary embolism is suspected.^46, 47^

Both elevated cardiac biomarkers and morphological signs for RV dysfunction portend a worse prognosis in patients with pulmonary embolism.^28, 48^ However, these signs are non-specific, and the role of biomarkers and/or echocardiographic assessment on a routine basis in all patients with acute pulmonary embolism is of unproven benefit.^49–51^ In either case, thrombolytic treatment is not recommended in haemodynamically stable patients since outcome data are confounded by excessive bleeding events. In case of suspected acute *cor pulmonale* or in the presence of CS, immediate echocardiography provides valuable information for further treatment.

Catheter-directed thrombolysis and/or thrombus aspiration appear to be safe techniques, but their efficacy above conventional thrombolysis remains unproven. Surgical embolectomy is a high-risk procedure that, according to guidelines, may be considered for high-risk PE in refractory CS or cardiac arrest.^22^ However, all these treatment options have revealed conflicting data without proven benefit for mortality, and evidence supporting these techniques remain sparse,^52–54^ limiting their use to experienced centres and ideally within a defined study/research setting. Only patients with a clear contraindication for anticoagulation should receive an inferior vena cava filter^55^ Even in these patients, the filter should be removed as soon as possible since the risk for the development of subsequent deep venous thrombosis is substantially elevated.^56^

Chronic right heart failure is, at least usually, the consequence of pulmonary hypertension, resulting in pathophysiological adaption of the RV.^17^ Chronic elevation of the pulmonary pressure is classified into five distinct clinical groups. Whilst PAH (WHO group 1) is rare, pulmonary hypertension secondary to left heart pathology (WHO group 2) or as a consequence of pulmonary disease (WHO group 3) is more common.^57^ In this context, it should be emphasized that current evidence precludes the routine use of specific vasodilators outside WHO group 1 (PAH) and WHO group 4 (chronic thrombo-embolic pulmonary hypertension, CTEPH). Management of these two complex entities should be exclusively undertaken in specialized centres with a multidisciplinary team (MDT) approach and high-end treatment options including the use of assist devices and access to transplantation. The use of specific vasodilating agents for all other groups of PH is not supported by the available published evidence. As such, treatment of acutely decompensated *cor pulmonale* should focus on supportive therapies that are directed against the underlying disease of the left heart and/or the lung. Since specific vasodilators might improve pulmonary haemodynamics but increasing ventilation/perfusion mismatch or shunting simultaneously results in worsening oxygenation, caution should be used regarding the implementation of vasodilating agents outside the recommendations of guidelines or studies. These treatment options should be considered strictly as off-label therapies and be used only as rescue therapy under close monitoring.

## Mechanical circulatory support for acute right ventricular failure

A range of mechanical circulatory support (MCS) devices are available to provide acute support via either direct or indirect bypass to the failing right heart, with the level of support and ability to provide oxygenation depending on the device selected.^58^ These include ECMO and its different configurations, the Impella RP and the Protek Duo, each of which have differing effects on the right and left circulation (*Table 2*).^59^ Insertion of an IABP is generally not recommended for isolated RV failure,^58^ and although historically extracorporeal CO_2_ removal has been used in acute RV failure with elevated PVR in the context of hypercarbia and/or lung transplantation, this has largely been superseded.

**Table 2:** Configuration and haemodynamic effects of different acute MCS support

Device	Configuration	Direct/indirect	Effect on haemodynamics	Oxygenation
VA-ECMO	Caval vein to descending aorta	indirect	RAP ↓	Yes
			PASP →	
			PA PP →	
			RVSV →	
			LV afterload ↑	
			LV systolic pressure ↑	
			LV SV ↓	
VVA-ECMO	Caval/femoral vein to descending aorta	indirect	RAP ↓	Yes ↑↓→
			PAMP ↓	
			RVSV ↓	
			LVEDP ↑ or ↓	
			LV systolic pressure ↑	
VAV-ECMO	Central vein to descending aorta and central vein	indirect	RAP may ↓	Yes
			PAMP may ↓	
			LVEDP ↑ or ↓	
			LV systolic pressure ↑	
Impella	Right atrium to pulmonary artery	direct	Native RV SV ↓	No
			RV systolic pressure ↑	
			PA systolic pressure ↑	
			PA pulse pressure ↓	
			RAP ↓	
			LVEDP ↑	
			LV SV ↑	
Protek Duo	Right atrium to pulmonary artery	direct	Native RV SV ↓	Yes
			RV systolic pressure ↑	
			PA systolic pressure ↑	
			PA pulse pressure ↓	
			RAP ↓	
			LVEDP ↑	
			LV SV ↑	

RV, right ventricle; SV, stroke volume; PA, pulmonary artery; RAP, right atrial pressure; LVEDP, left ventricular end-diastolic pressure; PASP, pulmonary artery systolic pressure; PAMP, pulmonary artery mean pressure; LV, left ventricle; VA, veno-arterial; VVA, veno-veno-arterial; VAV, veno-arterial-venous.

According to current guidelines, short-term MCS should be considered in patients with CS as bridge to recovery, bridge to decision, or bridge to bridge whilst the underlying causes for cardiogenic shock are addressed.^60^ Specifically, in case of pulmonary embolism, guidelines state that VA-ECMO may be considered in high-risk PE in combination with surgical embolectomy or catheter-directed treatment in refractory CS or cardiac arrest.^22^

Clinical parameters that suggest acute MCS use include signs of relative hypoperfusion plus haemodynamic features suggestive of RV failure (see above).^58^ A specific additional consideration relates to where acute left-sided MCS reveals acute RV failure. Discerning whether this is intrinsic RV failure or due to persistently elevated RV afterload from inadequate LV support defines ongoing management (escalation of LV support vs. introduction of RV support) can be determined by the use of the PA catheter demonstrating either an elevated or normal PVR.^61^ In general, the choice of device will be driven by the degree of support required including left/right ventricular interdependence, the clinical situation, the need for additional oxygenation, local expertise, and recommendations from the specialist MDT.^62^ As with all MCS, specialist cardiac critical care expertise is required for ongoing management.

## Conclusion

Acute *cor pulmonale* is a life-threatening condition involving several organ systems and frequently requiring coordinated delivery of care from all specialties involved, including emergency medicine, intensive care, cardiology, pneumology, and internal medicine. Understanding of the anatomical peculiarities of the RV and the pathophysiology leading to acute *cor pulmonale*, as well as an interdisciplinary consensus on the diagnostic modalities and therapeutic strategy, is mandatory for the optimal management of these patients (*Graphical abstract*).

## Data Availability

No new data were generated or analysed in support of this research.
